# Antiobesity, Antihyperglycemic, and Antidepressive Potentiality of Rice Fermented Food Through Modulation of Intestinal Microbiota

**DOI:** 10.3389/fmicb.2022.794503

**Published:** 2022-05-06

**Authors:** Papan Kumar Hor, Shilpee Pal, Joy Mondal, Suman Kumar Halder, Kuntal Ghosh, Sourav Santra, Mousumi Ray, Debabrata Goswami, Sudipta Chakrabarti, Somnath Singh, Sanjai K. Dwivedi, Miklós Takó, Debabrata Bera, Keshab Chandra Mondal

**Affiliations:** ^1^Department of Microbiology, Vidyasagar University, Midnapore, India; ^2^Bioinformatics Infrastructure Facility Center, Department of Microbiology, Vidyasagar University, Midnapore, India; ^3^Department of Biological Sciences, Midnapore City College, Paschim Medinipur, India; ^4^Division of Nutrition, Defense Institute of Physiology and Allied Sciences, New Delhi, India; ^5^Defence Research Laboratory (Defence Research and Development Organisation), Tezpur, India; ^6^Department of Microbiology, Faculty of Science and Informatics, University of Szeged, Szeged, Hungary; ^7^Department of Food Technology and Biochemical Engineering, Jadavpur University, Kolkata, India

**Keywords:** rice fermented food, antiobesity, antihyperglycemic, antidepressant (AD), gut flora

## Abstract

The present study has been aimed at evaluating the antiobesity, antihyperglycemic, and antidepressive potentials of *Asparagus racemosus* starter-based rice fermented foods. High-throughput NGS technology has revealed a number of bacterial genera in the prepared fermented rice, such as *Lactobacillus* (29.44%), *Brevundimonas* (16.21%), *Stenotrophomonas* (6.18%), *Pseudomonas* (3.11%), *Bacillus* (2.88%), and others (<2%). Eight-week administration of rice fermented food has increased food intake, whole-body weight, organ weight, different fat masses, serum lipid profiles, and histology of liver and adipose tissues in HFD-induced obese mice. In addition, upregulation of fatty acid oxidation and downregulation of adipocytogenesis- and lypogenesis-related genes along with the expression of their regulatory nuclear factors such as PPARα, PPARγ, PPARδ, and SREBP-1c have also been noted. Moreover, fermented food decreases fasting blood glucose level and improves glucose and insulin tolerance as well as the expression of GLUT4 receptor. Antiobesity and antihyperglycemic effects are also supported by the changes in insulin, leptin, and adiponectin hormone levels. The real-time polymerase chain reaction (RT-PCR) and denaturing gradient gel electrophoresis (DGGE) analyses have clearly demonstrated the intense colonization of Bacteroides, *Lactobacillus*, and *Bifidobacterium*, as well as the suppressed growth rate of γ- and δ-Proteobacteria and Firmicutes in the gut after fermented food intake. In the intestine, the latter group of microorganisms possibly modulate short-chain fatty acid (SCFA) levels such as acetate, butyrate, and propionate more than twofold. The impairment of memory-learning and anxiety-like obesity-associated cognitive phenotypes is mitigated significantly (*p* < 0.01) by fermented food as well. Thus, the formulated fermented food could be used as a natural therapeutic to alleviate obesity and its associated psychological and pathophysiological ailments.

## Introduction

Obesity is a complex multifactorial disease, where hereditary and metabolic factors interfere due to the gene–environment interaction. Literature suggests a strong correlation among obesity, type 2 diabetes (T2D), and mood alterations such as depression and neuropsychiatric disorders (Slyepchenko et al., 2016; Del Rocío Sevilla-González et al., 2017). Many epidemiological, clinical, and meta-analyses studies support the association of these three pathologies, leading to catastrophic complications, including both morbidity and mortality worldwide (Ning et al., 2020; González-Castro et al., 2021). A recent study has highlighted that obese individuals are 55% more likely to develop lifetime depression, while depressed individuals are 58% more likely to become obese than normal persons. Similarly, depression at baseline increases 37–60% type 2 diabetes risk (Milano et al., 2020). Obesity is linked to many pathophysiological conditions, and the most devastating are insulin resistance and the onset of type 2 diabetes (Barazzoni et al., 2018). In India, the economic reforms (1991) and subsequent over-powering have changed the quality of human lifestyle. Hyper-nutrition due to consumption of unhealthy energy-dense foods and a sedentary lifestyle might be linked to an increase in the prevalence of obesity and its comorbidities. According to recent studies, the prevalence rate of obesity varies from 11.8 to 31.3%, and within this range, 72 million people (8.8% of total population having age ≥ 18 years) are affected by diabetes in India (Ahirwar and Mondal, 2019; Gupta and Bansal, 2020). According to the Diabetes Foundation of India, about 80 million people will suffer from diabetes by 2025, making India the “Diabetes Capital of the world” (Gupta and Bansal, 2020). To date, pharmacological treatment (e.g., anorexic and fat absorption preventive drugs) does not appear as an effective measure for loss of body weight and its related pathophysiological states (Ray et al., 2018). The popular antidepressant drug fluoxetine has already become resistant to obese or overweight patients (Vagena et al., 2019).

Obesity, T2D, and depression, the intertwined trio metabolic syndromes are considered as the manifestation of chronic multisystem inflammation (Peirce and Alviña, 2019; Scheithauer et al., 2020). Diet-induced dysbiosis of intestinal microbiota (currently known as microbiota insufficiency syndrome) is changing the intestinal permeability (leaky gut) that is allowing the translocation of endotoxins (LPS) into systematics. This pathway is now hypothesized as a prime route for induction of chronic proinflammatory phenotype (Slyepchenko et al., 2016). The shift away from a “healthy” gut microbiome to decreased bacterial richness (dysbiosis) has promoted many intestinal and extraintestinal diseases (Slyepchenko et al., 2016). Conversely, colonization of healthy bacteria and their metabolic by-products, including some short-chain fatty acids (SCFAs), can play anti-inflammatory and immunomodulatory roles (Al Bander et al., 2020).

From ancient times, humans cultivated health-supporting microbes in fermented foods and beverages (Tamang et al., 2020, 2021; Tamang, 2021). The ingested food-graded healthy microbes, nutraceuticals, and bioactive metabolites in fermented food create a sociable environment in the gut that favors growth of good commensal bacteria and enhances their metabolic activities (Ray et al., 2016; Jha et al., 2019). For example, non-digestible carbohydrates (oligosaccharides/dietary fibers) in fermented food can effectively promote saccharolytic fermentation and synthesis of SCFAs such as acetate, propionate, and butyrate (approximate molar ratio of 60:20:20), which are considered as a master modulator of intestinal barrier functions, and have immunostimulatory, anti-inflammatory, glucose homeostasis, antiobesity, and many other health beneficial effects (Morrison and Preston, 2016; Al Bander et al., 2020). Preclinical lines of evidence strongly support the antiobesity effects of many isolated probiotic strains belonging to the genera *Lactobacillus* and *Bifidobacterium* (Kobyliak et al., 2018; Ray et al., 2018). Apart from isolated organisms, for the first time, it was advocated that Korean *kochujang*, a traditional fermented soybean-based red pepper paste, had significant antiobesity effects on human subjects (Lee et al., 2017). *Kimchi*, also a traditional Korean fermented vegetable food with lactic acid bacteria, exhibited antiobesity effects on mouse models (Han et al., 2015). In contrast to the isolated probiotic organisms, the matrix of fermented foods acts as a vehicle to deliver high number of live and bioactive microorganisms (multi-strain and multi-species) to the GI tract and protects them from the harsh environment of alimentary tract and, thus, promotes long-term survival of organisms in the gut (Ray et al., 2018; Jo et al., 2021).

Fermentation of food enhances nutritional and functional properties of food due to food–microbe interaction resulting in the transformation of substrates and formation of bioactive or bioavailable end-products that are beneficial for human health (Marco et al., 2017; Jha et al., 2019). Fermented food contains a diverse group of food-grade microorganisms, some of which are genetically similar to probiotics strains (Marco et al., 2017). Very recently, we have reported that *Asparagus racemosus*-based rice fermented food is a good source of lactic acid bacteria, *Bifidobacterium* sp., yeast, etc., enriched with lactic acid, acetic acid, adequate protein, fat, minerals, vitamins (water-soluble), oligosaccharide (G3-maltotriose), unsaturated fatty acids (ω3, ω6, ω7, and ω9), and a pool of essential and non-essential amino acids (Hor et al., 2021).

In this study, the healthiness of *A. racemosus* (rhizome)-based rice fermented food has been explored to establish the plant as an effective starter for traditional food preparation and its commercial usage. The main aim of this study is to examine the prophylactic effects of fermented rice against diet-induced obesity and its associated hyperglycemia and psychological disorders. Serological, hormonal, histological, psychological parameters, and the expression of some signature genes in a mouse model have been monitored and correlated with gut microbiota and their metabolites to determine the ability of prepared fermented food in regulation of obesity and its associated physiological consequences.

## Materials and Methods

### Plant Collection and Preparation of Fermented Food

The rhizome of *A. racemosus* was collected from natural forest of the Jangalmahal area of West Bengal, India, and was washed with sterile water and dried under sunlight. The root dust (0.5%, w/w) was further mixed with boiled rice and fermented at 37°C for 4 days (Hor et al., 2021). The fermented product was considered as a test sample (Ts). The unfermented control sample (Cs) was sterilized just after adding the starter into boiled rice to make it microbe-free.

### Analysis of Food Bacterial Community by Next-Generation Sequencing Methodology

Next-generation sequencing (NGS) technology is an emerging method and provides new insights into microbial diversity evaluation. For the NGS analysis, total community DNA of the sample was extracted using Qiagen (New Delhi, India) DNA kit method following the instructions. The quality of DNA was checked on 1% agarose gel, and the concentration was determined using a Nanodrop QC spectrophotometer (NanoDrop Technologies, Wilmington, DE, United States). Extracted pure DNA was subjected to PCR amplification targeting full-length 16S rDNA gene using 16F: 5′ AGAGTTTGATCMTGGCTCAG 3′; 16R: 5′ TACGGYTAC CTTGTTACGACTT 3′ primers. Each PCR reaction was performed for 28 cycles in a total volume of 25 μl by considering template-free reaction as a control. The PCR products (∼1,430-bp-long DNA fragments) were separated by agarose gel (1.5% w/v) electrophoresis. The target band was carefully excised from the gel and then purified using a QIA quick gel extraction kit (Qiagen, New Delhi, India) as per the manufacturer’s instructions. The final DNA pool was sent to the NGS facility of Bio-kart India Pvt Ltd. (Bangalore, India). Sequencing was performed using Oxford Nanopore Technologies (ONT MinION) (Jain et al., 2018). The ONT MinION is a portable sequencing device that generates maximum read lengths over 100 kb with the potential to span long repeats at comparably low cost and high speed (our test runs yielded 10–50 Gb in 48 h). The unassembled sequence data were analyzed using Metagenomics Rapid Annotation (MG-RAST) (v.4.0.3) online server^1^ comprising QIIME2 quality-based trimming. Generally, 16S rRNA sequence is extracted by MG-RAST with 70% identity using VSEARCH against a non-redundant database including SILVA, RDP, and Greengenes (Meyer et al., 2008). For our study, we opted SILVA SSU database for 16S rRNA extraction. The metadata was set for the V3–V4 region of 16S rRNA using MG-RAST for taxonomic classification of the bacteria present in the food samples. The high-throughput sequence data have been deposited in NCBI Sequence Read Archive (SRA) (BioProject ID: PRJNA691916 and BioSample ID: SAMN17308527).

### Animal Sorting and Treatment

Before conducting the animal experiment, ethical clearance was taken from the Institutional Ethics Committee, Vidyasagar University (IEC/7-9/C-9/16). Inbred male albino mice (15 ± 5 g) were collected and kept in a polypropylene cage under a standard atmospheric condition such as 12-h light/dark, 25 ± 5°C temperature, and 50 ± 5% humidity for 10 days. They were allowed to feed with a standard diet (Hindustan Lever, Mumbai, India) and sterile water *ad libitum*. The mice were randomly selected and assigned into four groups (*n* = 20) according to the type of diet and therapy: Group I (ND)—normal diet, mice maintained by providing procured feed; Group II (HFD)—high-fat diet (formulated lab-made feed); Group III (HFDC)—HFD along with control food sample (Cs); Group IV (HFDT)—HFD with test sample (Ts). The daily energy intake of the high-fat diet (HFD) group contributed 38.9% fat, 38.9% carbohydrate, and 22.2% protein (total 22.3 KJ/g), whereas normal diet (ND) contained 13.5% fat, 64.2% carbohydrate, and 22.3% protein (total 15.97 KJ/g). The HFDC and HFDT groups were fed 1.0 g (i.e., 20%, w/w) of control (Cs) and test samples (Ts), respectively, along with the HFD. Dose of fermented food was selected considering the quantity of viable microbes as per our previous experiment (Ray et al., 2018). The amount of food intake, body weight, and fecal bacteria was regularly monitored for 8 weeks. After completion of treatment, all the mice were euthanized by cervical dislocation, and different organs were excised and weighed. The blood samples were collected by cardiac puncture tested for biochemical analysis.

### Biochemical Analysis of Serum

The serum profile of total cholesterol (TC), triglycerides, high-density lipoprotein (HDL-C), low-density lipoprotein (LDL-C), very low-density lipoprotein (VLDL-C), alanine amino transferase (ALT), aspartate amino transferase (AST), total protein, and hemoglobin was analyzed using kit assay methods (Auto Span diagnostic kit, Mumbai) as per instruction of the manufacturer.

The serum levels of insulin, leptin, and adiponectin were determined by mouse-specific indirect ELISA kit assay (Abgenex, India) following the manufacturer’s direction.

### Glucose Tolerance Test and Insulin Tolerance Test

Blood glucose level was monitored using a glucose meter (Code-free, SD BIOSENSOR, India). For this experiment, mice were kept under fasting for 12–15 h, and then glucose solution (2 g/kg) was injected into intraperitoneal cavity. Blood glucose level was measured by collecting blood from the tail end of each mouse at an interval of 30 min gradually up to 150 min.

To conduct the insulin tolerance test (ITT), animals were fed at 1200 h. Human insulin was injected into all mice intraperitoneally (0.75 unit/kg of body weight). Blood glucose level was measured immediately before and after insulin injection. Results were expressed as percentage change in blood glucose level compared to initial level.

### Histological Examination of Liver and Epididymal Adipose Tissue

Thin sections of liver and epididymal adipose tissues were prepared by using Cryo-microtome (LEICA Biosystems Inc., CM1850, United States) and stained with Sudan-IV and hematoxylin-eosin, sequentially. The stained slides were observed under a microscope (Magnus invi, Olympus, India).

### Study of Genes Expression

The expression of targeted genes was studied as per our previous experiment (Ray et al., 2018). Briefly, fresh liver tissue of 100 mg was dissolved in 1 ml of TRIZOL reagent and then homogenized, followed by centrifugation at 12,000 rpm for 15 min at 4°C. The collected supernatant was mixed with 0.2 ml of chloroform and vortexed for 30 s and again centrifuged at 12,000 rpm for 15 min at 4°C. The upper aqueous phase of supernatant was collected and 0.5 ml of isopropanol was added to it and then kept at room temperature for 5 min, followed by centrifugation as per previous manner. After that, pellet was taken and 1 ml of 80% ethanol was mixed and centrifuged at 7,500 rpm for 5 min at 4°C. Then, pellet was air-dried and mixed with 20 μl of RNase-free water. From isolated mRNA, cDNA was synthesized by using cDNA synthesis kit (HiMedia, Mumbai, India). Amplification of cDNA was performed by Gene Amp PCR system ECO 96 (Himedia, Mumbai, India). The cDNA (10%) was employed for PCR amplification of different lipogenesis and lipolysis-related genes using a specific pair of primer (Supplementary Table 1). The reaction mixture of 20 μl consisted of a PCR master mix, cDNA as a template, forward primer, reverse primer, and water. Amplification was performed with an initial step at 94°C for 5 min, followed by 28 cycles (94°C for 30 s; 55–60°C for 45 s; 72°C for 1 min), and final elongation timer was set for 10 min at 72°C. After completion of amplification, 5 μl of the end product and 5 μl of loading buffer were mixed and loaded in agarose (2% w/v) for gel electrophoresis. The band density was then analyzed by GS-700 imaging densitometer (Gel doc system) with molecular analyst software (version 1.5; Bio-Rad Laboratories, CA, United States).

### Fecal Bacteriological Analysis Through Real-Time Polymerase Chain Reaction (RT-PCR) and Denaturing Gradient Gel Electrophoresis (DGGE)

Initially, DNA was extracted from fecal sample by phenol-chloroform method (Ott et al., 2004). Then, real-time PCR analysis was performed using SYBR green master mix (HiMedia, India). The 16S rRNA gene-specific primer pair used for this experiment targeting the phylum Firmicutes, sub-phyla γ- and δ-Proteobacteria, and the genera *Bacteroides*, *Lactobacillus*, and *Bifidobacterium* is depicted in Supplementary Table 1 (Layton et al., 2006; Delroisse et al., 2008; Yang et al., 2015). Universal 16S rRNA bacterial primer was used as an internal standard. The amplification program was carried out as follows: 1 cycle at 94°C for 10 s, 40 cycles at 94°C for 5 s, each annealing temperature was set for 30 s, and ended at 72°C for 30 s. From the last step of each cycle, fluorescent products were detected. The melting curve was obtained through increments of heat with continuous fluorescent collection (Applied Biosynthesis, Invitrogen, India). From the changes in melting curves, bacterial folds in fecal flora of different treated groups were analyzed.

For denaturing gradient gel electrophoresis (DGGE) analysis, approximately 340 bp (V3) of 16S rRNA gene was amplified using “*Lactobacillus* (LAB)”-specific bacterial primers (Supplementary Table 1), and that was anchored to a GC clamp at the 5′ end of the reverse sequence (Ghosh et al., 2021). PCR amplification was carried out following the methodology of Ghosh et al. (2021). DGGE was performed with a DCode electrophoresis system (Bio-Rad). A gradient gel (38–53%) of urea and formamide in a polyacrylamide gel (8%) was prepared, having the dimension of 16 cm × 16 cm × 1 mm. PCR product was used for gel electrophoresis at a constant temperature of 60°C and voltage of 60 V for 16 h. Gels were stained with ethidium bromide (0.5 mg/L) in TAE buffer for 20 min and then destained with sterile deionized water for 10 min, and viewed under UV transilluminator.

### Analysis of Short-Chain Fatty Acids in Intestinal Content

The method of Stein et al. (1992) was adopted with some modifications to determine SCFAs in intestinal content. Briefly, 1 g of intestinal content from each treated group was homogenized with 0.9% NaCl solution. Then, it was mixed with 10 ml of bovine serum albumin solution (5%) in separate conical tubes and vortexed for 2 min followed by centrifugation at 2,000 rpm for 10 min. One milliliter of supernatant from each group was collected in a round-bottomed flask, then acidified and frozen under a liquid nitrogen chamber. The volatile fatty acids were extracted under a vacuum and dissolved in alkaline solution. SCFA standards like acetate, butyrate, and propionate (Sigma, United States) were prepared in 5% BSA solution. The extracted SCFA samples and their corresponding standards were charged into the HPLC system (Agilent, United States), where the mobile phase was a combination of 0.2 M H_2_SO_4_ and acetonitrile solutions (75:25, v/v) with a flow rate of 0.8 ml/min. Both the samples and standards were injected at the volume of 10 μl, and column temperature was adjusted to 60°C and detected at a wavelength of 214 nm.

### Analysis of Psychological Behavior of Experimental Mice

Different maze tests were performed with experimental mice to assess the behavioral pattern as well as their learning-memory ability. This study was performed using Maze tests in a closed room with supplementation of standard environment by maintaining room temperature, air-conditioning, light–dark condition, etc. (Shoji et al., 2012; Aitta-Aho et al., 2016).

#### T-Maze Test

A “T” maze model was constructed using black painted paper wood consisting of one central straight arm and two-goal arms attached to the left and right directions of central arm, with dimensions of 0.4 × 0.1 m each and walls of 0.1-m height. The straight arm was marked as “A,” the left arm as “B,” and the right arm as “C.” Each mouse was placed in the central portion toward the “A” arm. Digital camera was established to record the behavior pattern of each mouse with videography. Traveled distance (m), time spent (s), and movement pattern of each mouse were calculated from the video recorded during the 30-min test period.

#### Elevated Plus Maze Test

An elevated plus-maze model was constructed as stated above. This model had four arms: two open arms and two closed arms. All the arms were connected to a central square (0.1 × 0.1 m). Arms of same types were located in opposite directions. The plus-maze setup was elevated 0.5 m above the floor. Each mouse of different treated groups was placed in the central square at the beginning of the experiment. Number of entries in each arm, total distance traveled (m), time spent (s), and movement pattern of each mouse in open and closed arms were calculated from video recorded during the 30-min test period.

### Statistical Analysis

All the data were presented as mean ± SD of a minimum of three replicas. One-way ANOVA was used to analyze significant differences and *t*-test was performed by taking all possible pairs using Sigma plot 11.0 (United States) statistical software.

## Results

### Evaluation of Bacterial Loads in Food Sample

**FIGURE 1:**
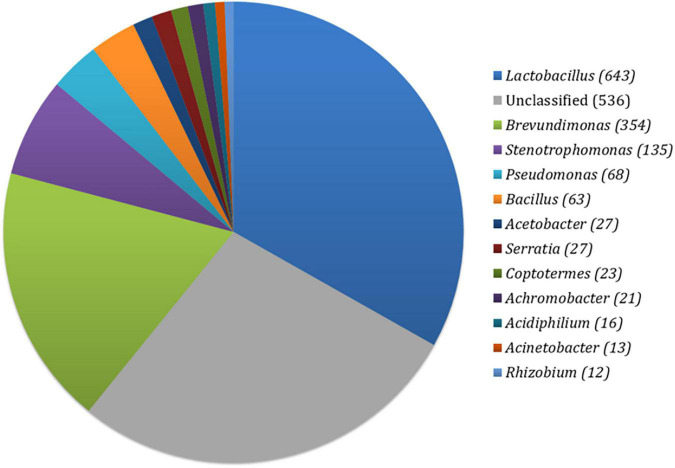
Distribution of bacterial OTUs in prepared fermented food.

In the prepared fermented food sample, a diverse range of bacterial taxa was observed using closed-reference OTUs picking method. Among them, a total of 643 (29.44%) OTUs belonged to the genus *Lactobacillus*, followed by *Brevundimonas* (354, 16.21%), *Stenotrophomonas* (135, 6.18%), *Pseudomonas* (68, 3.11%), *Bacillus* (63, 2.88%), *Acetobacter* (27, 1.24%), *Serratia* (27, 1.24%), *Coptotermes* (23, 1.05%), *Achromobacter* (21, 0.96%), *Acidiphillum* (16, 0.96%), *Acinetobacter* (13, 0.60%), and *Rhizobium* (12, 0.55%) (Figure 1). The rest of the OTUs were assigned as unclassified (536). Furthermore, the alpha diversity of bacterial community by calculating the Shannon (1.83) and Simpson (0.77) index implied that a higher range of species diversity as well as their abundance existed in our prepared fermented food sample.

### Effects of Rice Fermented Food on Physical and Serological Status of Experimental Mice

#### Body and Organ Weights

After administration of prepared fermented food, the body weight of all experimental animals/groups was increased compared to initial weight at the end of 8 weeks (Table 1A). The average body weight was increased by 13.05 g in ND, 24.48 g in HFD, 24.91 g in HFDC, and 17.31 g in the HFDT groups (Table 1B). The body mass index (BMI) was also increased with weight gain in respective groups. Rice fermented food supplementation in the HFDT group significantly (*p* < 0.001) diminished the organs’ weight such as liver, spleen, kidney, small intestine, and fat bodies like mesenteric, subcutaneous, perirenal, and epididymal in comparison to HFD and HFDC groups.

**TABLE 1A:** Analysis of food intake, body weight, and fat-deposited organ weight profile.

Parameters	ND	HFD	HFDC	HFDT
Food intake (g/mouse/day)	4.26 ± 0.25^a^	3.63 ± 0.11^c^	3.62 ± 0.31^c^	3.71 ± 0.19^c^
Initial body weight (g)	15.14 ± 0.15^a^	15.05 ± 0.51^a^	15.13 ± 0.74^a^	15.03 ± 0.68^a^
Final body weight (g)	28.19 ± 0.53^c^	39.53 ± 0.43^a^	40.04 ± 0.73^a^	32.34 ± 0.69^b^
Changes in body weight (g)	13.05 ± 0.63^d^	24.48 ± 0.32^b^	24.91 ± 0.36^a^	17.31 ± 1.02^c^
Initial body length (cm)	19.07 ± 0.99^a^	19.05 ± 0.71^a^	19.12 ± 0.18^a^	19.05 ± 1.00^a^
Final body length (cm)	19.39 ± 1.22^b^	20.84 ± 0.92^a^	20.76 ± 0.33^a^	19.98 ± 1.27^b^
Changes in body length (cm)	0.31 ± 0.23^c^	1.79 ± 0.14^a^	1.75 ± 0.21^a^	0.96 ± 0.35^b^
Initial BMI (g/cm^2^)	0.03	0.04	0.04	0.04
Final BMI (g/cm^2^)	0.07	0.09	0.09	0.08
Epididymal fat (mg/g body weight)	0.46 ± 0.04^c^	0.66 ± 0.05^a^	0.63 ± 0.03^a^	0.52 ± 0.03^b^
Subcutaneous fat pad (g)	0.29 ± 0.02^b^	0.57 ± 0.04^a^	0.56 ± 0.02^a^	0.31 ± 0.02^b^
Mesenteric fat pad (g)	0.17 ± 0.01^c^	0.38 ± 0.03^a^	0.38 ± 0.02^a^	0.24 ± 0.03^b^
Perirenal fat pad (g)	0.38 ± 0.03^c^	0.57 ± 0.04^a^	0.56 ± 0.04^a^	0.44 ± 0.02^b^
Liver (mg/g body weight)	1.82 ± 0.02^b^	2.49 ± 0.06^a^	2.50 ± 0.01^a^	1.78 ± 0.02^c^
Kidney (mg/g body weight)	0.38 ± 0.02^c^	0.63 ± 0.03^a^	0.63 ± 0.03^a^	0.45 ± 0.03^b^
Spleen (mg/g body weight)	0.19 ± 0.02^c^	0.35 ± 0.03^a^	0.36 ± 0.03^a^	0.25 ± 0.03^b^
Small intestine (mg/g body weight)	1.05 ± 0.05^c^	1.52 ± 0.02^a^	1.53 ± 0.06^a^	1.35 ± 0.05^b^

*All parameters are presented here as mean ± standard deviation (n = 3).*

*The values without common elementary knowledge are significantly different (p < 0.001) from each other and are shown in superscript letters in different rows.*

**TABLE 1B:** The body weight and food intake details in each week.

Time	Parameters	ND	HFD	HFDC	HFDT
1st Week	Food intake (g/mouse/day)	3.98 ± 0.45^a^	3.49 ± 0.28^b^	3.55 ± 0.12^b^	3.37 ± 0.42^c^
	Initial body weight (g)	15.14 ± 0.15^a^	15.05 ± 0.51^a^	15.13 ± 0.74^a^	15.03 ± 0.68^a^
	Final body weight (g)	16.21 ± 0.25^c^	19.74 ± 0.24^a^	19.79 ± 052^a^	17.92 ± 0.78^b^
2nd Week	Food intake (g/mouse/day)	3.45 ± 0.15^c^	3.57 ± 0.32^b^	3.55 ± 0.38^b^	3.72 ± 0.25^a^
	Initial body weight (g)	16.21 ± 0.25^c^	19.74 ± 0.24^a^	19.79 ± 052^a^	17.92 ± 0.78^b^
	Final body weight (g)	18.34 ± 0.45^c^	23.54 ± 0.46^a^	23.58 ± 0.75^a^	19.30 ± 0.58^b^
3rd Week	Food intake (g/mouse/day)	4.32 ± 0.16^a^	3.63 ± 0.34^c^	3.69 ± 0.25^cb^	3.75 ± 0.17^b^
	Initial body weight (g)	18.34 ± 0.45^c^	23.54 ± 0.46^a^	23.58 ± 0.75^a^	19.30 ± 0.58^b^
	Final body weight (g)	19.87 ± 0.36^d^	23.65 ± 0.24^b^	24.01 ± 054^a^	20.85 ± 0.38^c^
4th Week	Food intake (g/mouse/day)	4.12 ± 0.24^a^	3.62 ± 0.32^c^	3.67 ± 0.12^c^	3.85 ± 0.65^b^
	Initial body weight (g)	19.87 ± 0.36^d^	23.65 ± 0.24^b^	24.01 ± 054^a^	20.85 ± 0.38^c^
	Final body weight (g)	20.32 ± 0.34^c^	26.34 ± 0.12^a^	26.28 ± 0.22^a^	22.35 ± 0.12^b^
5th Week	Food intake (g/mouse/day)	4.47 ± 0.28^a^	3.72 ± 0.45^c^	3.69 ± 0.45^c^	3.84 ± 0.22^b^
	Initial body weight (g)	20.32 ± 0.34^c^	26.34 ± 0.12^a^	26.28 ± 0.22^a^	22.35 ± 0.12^b^
	Final body weight (g)	22.38 ± 0.35^c^	28.75 ± 0.77^a^	28.81 ± 0.82^a^	25.12 ± 0.38^b^
6th Week	Food intake (g/mouse/day)	4.45 ± 0.28^a^	3.62 ± 0.32^c^	3.61 ± 0.12^c^	3.75 ± 0.25^b^
	Initial body weight (g)	22.38 ± 0.35^c^	28.75 ± 0.77^a^	28.81 ± 0.82^a^	25.12 ± 0.38^b^
	Final body weight (g)	24.86 ± 0.24^d^	32.56 ± 0.65^a^	32.62 ± 0.72^a^	28.56 ± 0.32^c^
7th Week	Food intake (g/mouse/day)	4.55 ± 0.27^a^	3.67 ± 0.56^cb^	3.61 ± 0.32^dc^	3.74 ± 0.54^b^
	Initial body weight (g)	24.86 ± 0.24^d^	32.56 ± 0.65^a^	32.62 ± 0.72^a^	28.56 ± 0.32^c^
	Final body weight (g)	26.98 ± 0.46^c^	36.15 ± 0.52^a^	36.78 ± 0.48^a^	30.86 ± 0.76^b^
8th Week	Food intake (g/mouse/day)	4.75 ± 0.32^a^	3.75 ± 0.26^b^	3.78 ± 0.56^b^	3.77 ± 0.28^b^
	Initial body weight (g)	26.98 ± 0.46^c^	36.15 ± 0.52^a^	36.78 ± 0.48^a^	30.86 ± 0.76^b^
	Final body weight (g)	28.19 ± 0.53^c^	39.53 ± 0.43^a^	40.04 ± 0.73^a^	32.34 ± 0.69^b^

*All parameters are presented here as mean ± standard deviation (n = 3).*

*Data with different superscript letters are significantly different (p < 0.001) from each other in their respective row.*

#### Serum Lipid Profile

A significant abatement of serum level of cholesterol (30.99%), triglyceride (28.39%), and other related parameters was noted in the HFDT group compared to HFDC or HFD (Table 2). Ratios of HDL/cholesterol and HDL/LDL were also significantly enhanced in the HFDT group.

**TABLE 2:** Serum profiling of different experimental groups.

Parameters	ND	HFD	HFDC	HFDT
Total sugar (mg/dl)	117.22 ± 2.78^c^	203.29 ± 5.59^a^	202.02 ± 6.18^a^	145.72 ± 6.92^b^
ALT (U/L)	47.79 ± 2.88^c^	65.17 ± 3.35^a^	64.84 ± 4.09^a^	52.61 ± 2.33^b^
AST (U/L)	44.86 ± 2.79^c^	67.16 ± 4.20^a^	67.29 ± 1.23^a^	50.62 ± 3.83^b^
Hemoglobin (mg/dl)	10.57 ± 0.47^c^	16.13 ± 1.45^a^	15.83 ± 1.09^a^	13.75 ± 0.37^b^
Total protein (g/dl)	3.12 ± 0.02^a^	1.92 ± 0.02^c^	1.93 ± 0.03^c^	2.55 ± 0.10^b^
Total cholesterol (mg/dl)	137.75 ± 3.52^c^	216.39 ± 5.48^a^	217.01 ± 6.28^a^	149.33 ± 1.16^b^
Triglyceride (mg/dl)	165.23 ± 2.73^c^	301.37 ± 3.12^a^	301.15 ± 6.84^a^	215.61 ± 4.26^b^
HDL-c (mg/dl)	52.57 ± 1.59^b^	35.73 ± 2.79^c^	34.62 ± 3.23^c^	75.31 ± 4.21^a^
LDL-c (mg/dl)	71.24 ± 1.19^c^	159.71 ± 2.34^a^	160.03 ± 5.33^a^	122.99 ± 2.29^b^
VLDL-c (mg/dl)	33.04 ± 0.54^c^	60.27 ± 0.62^a^	60.22 ± 1.37^a^	43.11 ± 0.85^b^
Total cholesterol/HDL	2.61 ± 0.09^b^	6.07 ± 0.31^a^	6.32 ± 0.71^a^	1.98 ± 0.12^c^
HDL/LDL	0.73 ± 0.01^a^	0.216 ± 0.01^c^	0.21 ± 0.01^c^	0.51 ± 0.12^b^
Insulin (pg/ml)	2309.33 ± 55.85^c^	5743 ± 104.27^a^	5740 ± 63.76^a^	3731 ± 89.37^b^
Leptin (pg/ml)	2304 ± 130.01^c^	4479.667 ± 36.05^a^	4468 ± 91.24^a^	3279 ± 115.70^b^
Adiponectin (ng/ml)	14.79 ± 1.86^b^	7.47 ± 0.37^c^	7.60 ± 0.22^c^	33.79 ± 1.58^a^

*All parameters are presented here as mean ± standard deviation (n = 3).*

*The values without common elementary knowledge are significantly different (p < 0.001) from each other and are shown in superscript letters in different rows.*

#### Glucose and Insulin Tolerance Test

In the HFD group, blood glucose level was higher (>86.07 mg/dl), which indicated the prevalence rate of hyperglycemia state in them. However, glucose level was significantly lower in HFDT (>57.57 mg/dl) than HFD, whereas in the HFDC group, sugar levels remained higher than HFD (Table 2).

**FIGURE 2:**
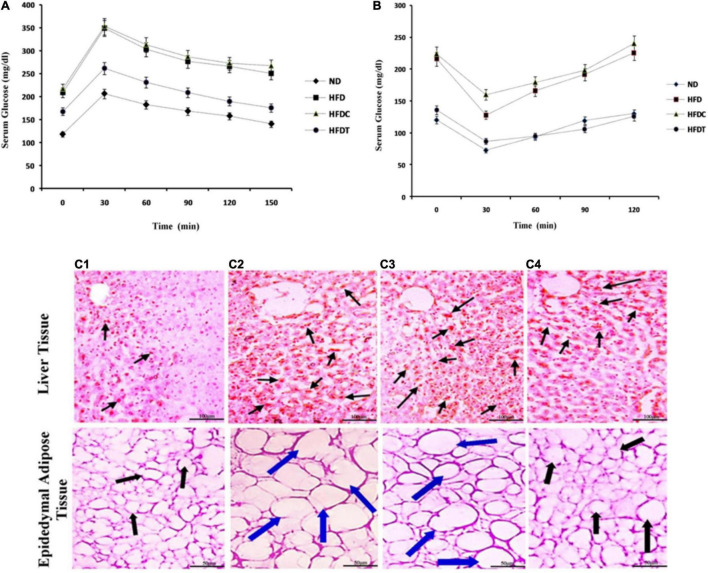
The effect of fermented product and its capacity of glucose homeostatis by glucose tolerance test (GTT, **A**) and insulin tolerance test (ITT, **B**) in ND, HFD, HFDC, and HFDT groups, respectively. Data are represented as mean ± SD of three replicates. In both cases significant differences of serum blood glucose levels are present between ND and HFD as well as between HFD and HFDT groups. No significant difference is present between HFD and HFDC groups. The effect of rice fermented beverage supplementation on liver and epididymal adipose tissue histoarchitecture **(C)**. Liver slice and epidedymal adipose tissue of mice fed with normal diet **(C1)**, high fat diet (HFD; **C2**), HFD supplemented with Unfermented product **(C3)**, and HFD with fermented rice product **(C4)** are stained with Oil red 0 (Liver) and hematoxylin and eosin (Adipose tissue). In case of liver tissues, arrows indicate deposited fat droplets in epididymal adipose tissues. Black arrow indicates normal diameter; blue arrow indicates increasing diameter of adipose tissues among different mice groups. For liver tissues scale bar is set at 100 μm, in contrary the scale bar for adipose tissues is set at 50 μm.

Glucose tolerance was significantly impaired in the HFD group, as shown by sustained high levels of blood glucose (delayed time-course of glucose clearance). Glucose intolerance in the HFD group was mitigated by supplementation of fermented food (HFDT group). However, no such notable effect was found in the HFDC group as compared to HFD (Figure 2A).

The ITT revealed that blood glucose level remained higher in both HFD and HFDC groups after 120 min of insulin injection, whereas the blood glucose level was normal in the HFDT group (Figure 2B).

#### Insulin, Leptin, and Adiponectin Level

The serum levels of insulin and leptin increased approximately twofold in HFD and HFDC compared to the ND group (Table 2). This indicated severe insulin resistance in the HFD group. However, significant diminution of insulin (35.05%) and leptin (26.61%) levels was noted in HFDT compared to the HFD group, whereas serum level of adiponectin decreased in HFD (49.49%) and HFDC (48.61%) groups in comparison to ND (Table 2). The adiponectin level improved (77.89%) in the HFDT group compared to HFD.

### Assessment of Liver Functions

The serum levels of alanine transaminase (ALT) and aspartate transaminase (AST) are reliable indicators for accessing liver function. It was noted that serum levels of both enzymes increased up to 17.38 and 22.3 U/L, respectively, in the HFD group than in ND (Table 2). A comparatively higher hemoglobin level was noted in both HFD and HFDC groups (Table 2). Notable reductions of serum ALT (12.56 U/L), AST (46.54 U/L), and hemoglobin (2.38 mg/dl) levels were observed in the case of HFDT compared to the HFD group. The impairment of liver functions in the HFD group was also reflected by diminished serum protein level (1.2 g/dl), which was replenished by supplementation of fermented foods (Table 2).

### Histological Examination of Liver and Adipose Tissue

Histological study demonstrated the accumulation of fat droplets (fatty appearance), hepatocytes, hypertrophy, and fibrotic liver changes in the HFD group (Figure 2C). Similarly, in adipose tissues, adipocytes were in hypertrophy state due to accumulation of fat and hyperplasia. Their changes were significantly attenuated in the HFDT group, as observed in the ND group (Figure 2C).

### Study of mRNA Expression of Fatty Acid Metabolism-Related Genes

**FIGURE 3:**
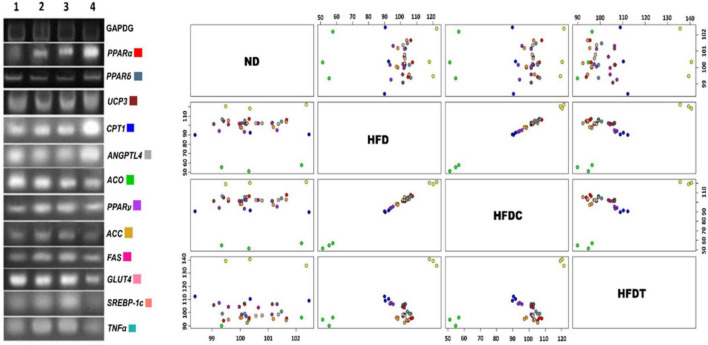
The mRNA expressions of obesity related (lipolysis and lipogenesis) genes isolated from mouse liver sample of different experimental groups. Lanes are marked as lane 1–ND, 2–HFD, 3–HFDC, and 4– HFDT. GAPDH has been used as a relative standard. Scatter plot matrix shows expression of genes: *PPAR*α, *PPAR*δ, *PPAR*γ, *ACC, ACO, UCP3, ANGPTL4, CPT1, FAS, GLUT4, SREBP-1c*, and *TNF*α. The color coding is indicating respective genes.

The relative expression of obesity-related genes in liver tissues was investigated to evaluate whether the reduced lipid accumulation in fermented food treatment (in HFDT group) was due to inhibition of adipocyte differentiation or alteration of lipid metabolism circuit (Figure 3). The level of expression of lipolysis regulatory transcription factor-like peroxisome proliferator-activated receptor α (PPARα), PPARδ, and their downstream products of fatty acid oxidation such as carnitine palmitoyltransferase 1 (CPT1), uncoupling protein 3 (UCP3), and glucose transporter 4 (GLUT4) were relatively higher in the HFDT group than HFD and HFDC. On the contrary, relative expressions of adipocytogenesis and fatty acid synthesis-related genes such as PPARγ, sterol regulatory element-binding protein-1c (SREBP-1c), acetyl-CoA carboxylase (ACC), and fatty acid synthase (FAS) were also reduced in HFDT than HFD and HFDC groups. It was also noted that expressions of inflammatory marker proteins such as tumor necrosis factor α (TNFα) and angiopoietin-like protein 4 (ANGPTL4) were also improved significantly in the HFDT group than HFD.

### Analysis of Fecal Flora

**FIGURE 4:**
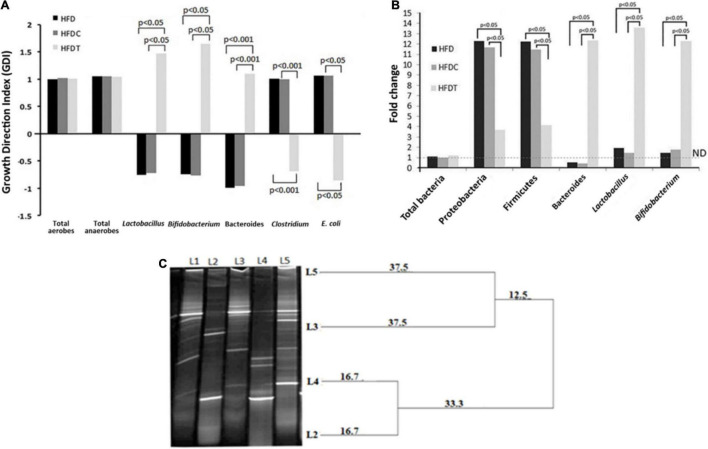
Changes in fecal microbial composition (analyzed by selective media) among different treatment groups compared with control group given as growth direction index (GDI) plot (base line at “0” scale). Data, shown as mean ± standard deviation of three replicates are significantly different at *p* < 0.05 and *p* < 0.001, where HFD, high fat diet; HFDC, high fat diet with control; and HFDT, high fat diet supplemented with fermented rice product **(A)**. Changes in fecal microbial composition among different treatment groups compared with control group are given as fold changes, analyzed through RT-PCR technique, and significance difference is present at *p* < 0.05 **(B)**. DGGE based profiling of LAB [Ll–marker *Lactobacillus* (*Lactoba cillus plantarum, Lactobacillus johnsonii, Lactobacillus criepatus, Lactobacillus lactis, Lactobacillus rhamnosus* from top to bottom), L2–plant residue, L3–rat fecal matter after consumption of plant residue, L4–fermented food with herbal starter, L5–rat fecal matter after consumption of fermented food]. The dendrogram is prepared based on analysis of similarity score and cluster from DGGE bands by PyElph software **(C)**.

The quantity of indicator culturable fecal bacteria in HFD, HFDC, and HFDT was enumerated. Their growth direction index (GDI) was calculated with respect to the control (ND) group (Figure 4A). Ecological dysbiosis of gut flora composition was established in the HFD group by revealing the enhancement of total anaerobes and aerobes, *Clostridium perfringens* (presenting organism in the phylum of Firmicutes), and *Escherichia coli*, but the decline of *Bifidobacterium* sp*., Lactobacillus* sp., and Bacteroides in comparison to the ND group. However, a significant improvement and establishment of healthier flora were noted with treatment of fermented food to the HFD-induced obese mice. The most notable changes were increment of *Bifidobacterium* sp. (1.42-fold) and *Lactobacillus* sp. (1.94-fold) and shifting of the ratio of *C. perfringens*: Bacteroides from 0.83 to 0.78 in the HFDT group compared to HFD.

### Comparison of Indicator Fecal Flora by Real-Time Polymerase Chain Reaction Analysis

The real-time polymerase chain reaction (RT-PCR) analysis demonstrated no significant alteration in total bacterial content of all treated groups in comparison to the ND group (Figure 4B). However, fermented food has a significant impact on indicator gut flora content, such as γ- and δ-Proteobacteria and Firmicutes, which expanded more than 10-fold in the HFD group, and their proliferation was significantly decreased in HFDT (seven to eightfold). Moreover, fermented food treatment significantly increased the colonization of the so-called health-beneficial bacterial genera such as Bacteroides, *Lactobacillus*, and *Bifidobacterium* in the HFDT group than HFD. However, compositional variation in HFDC group was very similar to the HFD group.

### Evaluation of Bacterial Succession From Food to the Gut by Polymerase Chain Reaction-Denaturing Gradient Gel Electrophoresis

DGGE analysis revealed the presence of a diverse group of LAB in plant residues of *A. racemosus* (rhizome), plant residue-induced rice fermented food, fecal content of only plant product consumed mice, and fecal content of fermented food consumed mice (Figure 4C). For the first time, our analysis highlighted the abundance of LAB in the experimented plant materials and their translocation into fermented food. The DGGE analysis also revealed the ecological succession and selection of specific bacterial community from plant material or fermented food into the intestine.

### Short-Chain Fatty Acids

The levels of SCFAs such as acetate, butyrate, and propionate (Table 3) revealed higher concentration of acetic acid than propionic and butyric acids in intestines of different treated groups. However, the amount was increased by twofold in the HFDT group than the HFD and HFDC groups. The amount of propionic and butyric acids was significantly decreased in HFD and HFDC groups than ND, whereas in HFDT, their concentrations were increased by twofolds.

**TABLE 3:** Analysis of short-chain fatty acids extracted from intestinal loop of treated groups (μg/ml).

Parameters	ND	HFD	HFDC	HFDT
Acetate	746.70 ± 31.67^b^	548.33 ± 37.49^d^	572.27 ± 39.11^c^	1263.04 ± 33.16^a^
Propionate	445.35 ± 39.99^d^	379.00 ± 28.48^c^	410 ± 30.41^b^	794.41 ± 26.47^a^
Butyrate	356.96 ± 43.08^b^	253.49 ± 38.41^d^	299.93 ± 13.47^c^	734.98 ± 26.52^a^

*All the parameters are presented here as mean ± standard deviation (n = 3).*

*The values without common elementary knowledge are significantly different (p < 0.05) from each other and are shown in superscript letters in different rows.*

### Psychological Behavior Examination

#### T-Maze Test

**FIGURE 5:**
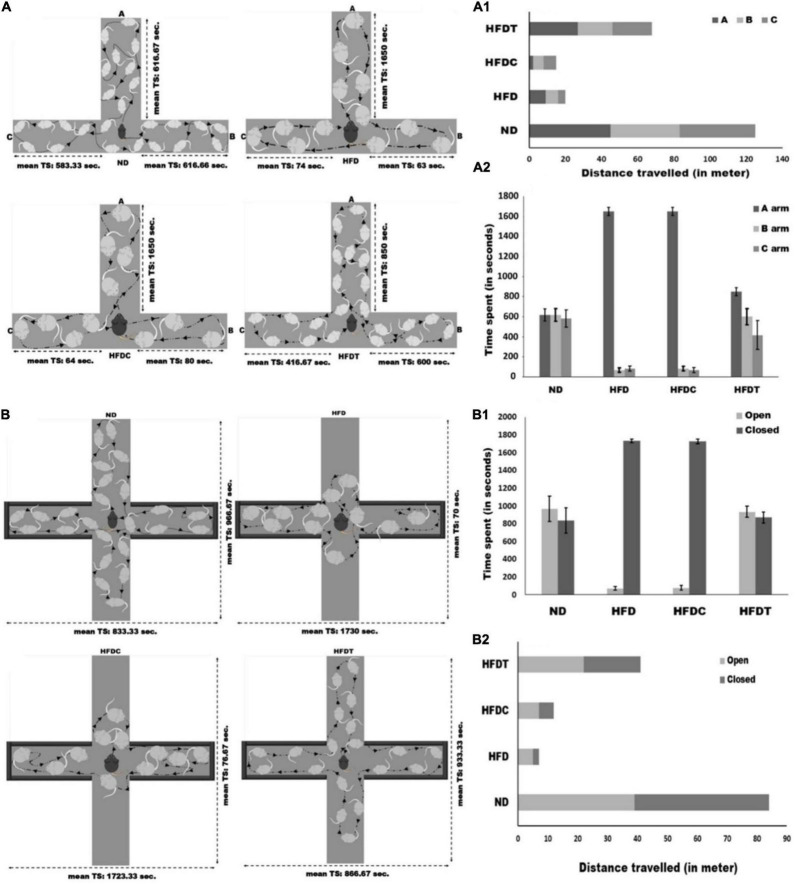
Schematic representation of *T-maze* experimental assay between different treated mice groups **(A)**. Total distance travelled and time spends in each arm of *T-maze* for different experimental groups are represented in **(A_1_, A_2_)**, receptively. Schematic representation of elevated *plus-maze* between different treated mice groups **(B)**. Total distance traveled and time spends in each open and close arms of elevated *plus-maze* for different experimental mice groups are represented in **(B_1_, B_2_)**, receptively. Data are represented as mean ± standard deviation of three replicates.

The T-maze test demonstrated different variables such as movement pattern, total distance traveled, and time spent of the mice in each arm. These are considered as useful indices to characterize the learning and memory ability of experimental mice groups. It was observed that mice of the ND group traveled maximum distance (125 m) in comparison to other treated groups (*p* < 0.01), whereas HFDT diet feed mice traveled 29.41 and 22.06% higher (*p* < 0.01) distance than HFD and HFDC groups, respectively (Figure 5). In case of time spent study, it was observed that mice of ND and HFDT groups spent relatively equal time in each arm, and no significant difference was found. However, maximum rest time was observed for HFD (1,600 ± 40.82 s) and HFDC (1,650 ± 39.62 s) groups of mice in “A” (starting) arm, which was significantly higher (*p* < 0.05) than HFDT and ND groups within a fixed time interval of 1800 s (Figure 5A).

#### Elevated Plus Maze Test

The elevated plus-maze test was performed to analyze some specific parameters such as time spent in open and closed arms, total distance traveled, and patterns of movement, which further illustrated the level of anxiety in all treated mice groups. Mice of ND and HFDT groups spent relatively equal time in both open and closed arms. Mice of HFD (1,730 ± 21.60 s) and HFDC (1,723.33 ± 24.94 s) groups spent maximum time in closed arms, which was significantly higher (*p* < 0.01) than HFDT and ND groups. Consequently, a higher distance (*p* < 0.01) was traveled by mice of HFDT group than HFD (17.03%) and HFDC (29.26%). However, mice of the ND group traveled maximum distance, i.e., 84 m through open and closed arms (Figure 5B).

## Discussion

From the dawn of human civilization, people have been using selective plant parts from their surroundings as food, medicine, and for other daily amenities. Supplementation of plant residues to prepare different fermented foods/beverages is a traditional practice in Asian people (Jha et al., 2019). In recent years, the exploration of fermented foods with their functional potentials to alleviate certain diseases/symptoms is drawing the attention of researchers in order to develop novel products leading to provide a sustainable human life. This type of process is now known as bio-prospecting. However, at the same time, it is noteworthy to mention that human physiology is very complex. Kobyliak et al. (2018) mentioned that a single molecule is unlikely to cure multifactorial diseases such as obesity, diabetes, and other metabolic disorders. The fermented foods could compensate the gap for containing a versatile group of effective nutraceuticals whose safety has been experienced by native people for generations (Hor et al., 2019).

Considering the perspectives, in this present research, a fermented rice product has been prepared by using the root dust of *A. racemosus* as a starter component, and its antiobesity potentials have been evaluated using animal models. Only 20% (w/w) of fermented food was supplemented along with animal diet, and this dose was selected on the basis of a number of viable microbes and to nullify the impact of nutrients. The newly formulated food consisted of a number of microorganisms such as mold, yeast, and a diverse range of bacterial genera (Hor et al., 2021). High-throughput metataxonomics analysis of 16S rRNA genes revealed a quite high range of bacterial diversity in our prepared fermented food sample with the most abundant OTUs of *Lactobacillus* sp. (29.44%). Bergsveinson et al. (2017) reported lactic acid bacteria as the predominant microbe in plant-based beverages produced from cereals and legumes by applying NGS/omics technology. The healthiness of *Lactobacillus* sp. is well characterized and they have also “generally regarded as safe (GRAS)” status. Herbal starter-based rice fermented beverage “*haria*” also contains anaerobic and microaerophilic microorganisms such as LAB, bifidobacteria, and yeast (Ghosh et al., 2021), favorable microorganisms for human health.

The supplementation of fermented rice in parallel with the HFD significantly improved the physical and structural phenotypes in obese mice, including body weight, BMI, different organ weights, and weights of different deposited fat bodies (Table 1A). It alluded that fermented food could alleviate fat accumulation in different body parts and thereby reducing total body weight by about 26% with respect to diet-induced obese mice (HFD group) after 8 weeks of treatment. Though the dietary intervention and its *modus operandi* on experimental mice are distinct from a human subject, our result is more consistent with recommended weight reduction (5–10%) by the US National Heart, Lung, and Blood Institute (NHLBI Obesity Education Initiative Expert Panel on the Identification, 1998), the WHO (2000), and the Canadian Obesity Guidelines to reduce health risk from chronic disease (Strychar, 2006).

The characteristics of dyslipidemia, hypertriglyceridemia, and hypercholesterolemia in the HFD group were partially mitigated by supplementation of fermented food. These serological indicators are major risk factors associated with cardiovascular diseases (CVDs) and obesity (Vecchié et al., 2018). Lowering of serum triglycerides and cholesterol levels is also linked to enhancement of insulin resistance and blood glucose level in HFDT. Moreover, the HDL-C level, regarded as good cholesterol for facilitating LDL-C elimination, was also significantly increased in the HFDT group (Table 2).

Furthermore, glucose and insulin intolerance were noted in HFD and HFDC groups. The plasma level of insulin and leptin remained very high in HFD and HFDC; however, adiponectin level was lower in those groups than in ND and HFDT. In HFD mice, the symptoms of insulin resistance, hyperinsulinemia, hypertriglyceridemia, and hyperglycemia might be associated with obesity and type 2 diabetes-like metabolic syndromes. Accumulation of fatty acids and their metabolites (diacylglycerol) in cells was attributed for being induced by non-responsiveness of insulin receptors *via* the protein kinase C (PKC) pathway. Activated PKCδ generally alters tyrosine phosphorylation of insulin receptor substrate 1 (IRS1) and prevents the insulin signaling pathway, resulting in a decrease in translocation of glucose transporters into plasma membrane, and thus increases insulin resistance (Samuel et al., 2010; Balsan et al., 2015). Furthermore, in the liver, plasma-free fatty acids might increase glucose synthesis (gluconeogenesis) and decrease insulin clearance, thus amend the effect of insulin resistance. It has been reported by Perry et al. (2016) that a fatty diet can elevate gut flora-derived acetate, which stimulates the parasympathetic nervous system to overproduce insulin from β-cells as well as increase the level of hunger-associated hormone ghrelin resulting in a vicious cycle, which promotes overfeeding of fat and thus disrupts glucose homeostasis. The high insulin level in blood generally upregulates lipogenic enzymes and promotes fat deposition in adipose tissues (Balsan et al., 2015; Perry et al., 2016). In this context, our results also demonstrated HFD-induced hyperplasia and hypertrophy of adipocytes resulting in higher leptin secretion from fatty adipose tissues, which might cause hyperleptinemia (Unger, 2004). A significant negative correlation was observed among plasma level of adiponectin and BMI, blood glucose, insulin, triglyceride level, as well as visceral fat accumulation. Moreover, insulin resistance also decreases the expression of adiponectin receptor in adipose tissues and thus impairs autocrine action of adiponectin resulting in the inhibition of adiponectin synthesis in obese patients (Balsan et al., 2015).

We observed that supplementation of fermented food (HFDT) led to a significant and perceptible improvement of hyperglycemia, glucose tolerance, levels of glucose metabolism-related hormones in experimental mice (Table 2). These results indicated that nutraceuticals-rich fermented food might play an important role in lowering down dyslipidemia, particularly triglyceride and LDL-C/VLDL-C levels, which might lessen insulin resistance, thus enhancing glucose catabolism. Enhanced level of insulin sensitivity and glucose tolerance was also reflected by higher expression of GLUT4 receptors in liver cells of HFDT than that of the HFD and HFDC groups.

The hepatocytes in liver play a key role in lipid metabolism by proficiently up-taking non-esterified fatty acids (from adipose tissue) through its specific channel [fatty acid transport protein (FATP) or fatty acid translocase (FAT)], thus facilitating fatty acid synthesis and esterification (“input”) as well as fatty acid oxidation and triacylglycerol export *via* VLDL (“output”) (Jensen-Urstad and Semenkovich, 2012). The acceleration of lipogenesis which led to the deposition of fat droplets in liver was reflected by histological studies in the HFD group. Fatty liver or hepatic steatosis is a common cause of jaundice, ascites, and edema (Jensen-Urstad and Semenkovich, 2012). Amelioration of HFD-induced structural and functional deformity of liver by supplementation of fermented foods accompanied significant improvement in SGOT, SGPT, hemoglobin levels, and overall histoarchitecture (Table 2 and Figure 2C).

In recent years, obesity has been recognized as a chronic and systemic inflammatory disease (dietary fat-induced leaky gut that leads to fastening of LPS diffusion and induced systemic pro-inflammatory states) and liver is the worst affected organ (Slyepchenko et al., 2016). It was observed that expression of TNF-α was significantly lower in HFDT than in HFD and HFDC groups (Figure 3). It prominently indicated the mitigation of systemic inflammatory responses (by an unknown mechanism) by applying fermented food treatment.

From the metabolic point of view, obesity is a result of increased fatty acid synthesis and decreased oxidation of fat or low energy expenditure from fat. In our experiment, to evaluate the *modus operandi* of anti-obese fermented food, we measured relative mRNA expression of genes coding enzymes associated with fatty acid metabolism and their related transcriptional factors, i.e., peroxisome proliferator-activated receptors (PPARs) and sterol regulatory element-binding protein (SREBP 1c), which were reported as master regulators of different genes associated with fat metabolism and adipocytogenesis (Varga et al., 2011). A significant upregulation of the expressions of *PPAR*α, *PPAR*δ, and their downstream products such as ACO, UCP3, CPT1, and ANGPTL4 indicated the enhancement of lipolysis in an HFDT group than HFD (Figure 4). In contrast, the downregulation of SREBP-1c, PPARγ, ACC, and FAS led to curtailment of adipocytogenesis and lipogenesis was observed by the supplementation of fermented food (HFDT). This study demonstrated that fermented food could accelerate reciprocal regulation of both fatty acid breakdown (upregulation ACO and CPT1, the rate-limiting enzymes of β-oxidation) and biosynthesis (downregulation of FAS, the master enzyme of fatty acid synthesis) (Ray et al., 2018). Improvement of insulin sensitivity and unsaturated fatty acids (in fermented food) might enhance the expression of PPARα in the HFDT group (Samuel et al., 2010), thus upregulating the uncoupling protein (UCP3), a family of mitochondrial transporters, which facilitated fatty acid export from matrix site and protected mitochondria from lipid-induced oxidative damages in HFDT (Acosta et al., 2015). The ANGPTL4 is a circulating lipoprotein lipase inhibitor that promotes plasma TG clearance and decreases liver TG synthesis (Koliwad et al., 2012). It was observed that our prepared fermented food upregulated the expression of ANGPTL4, a glucocorticoid-dependent fatty acid gate keeper (Koliwad et al., 2012), resulting in an improvement of glucocorticoid level in the HFDT group.

Diet-induced alterations of gut microbiota are reported to change in relative abundance; therefore, selective culture-based or strain-specific qPCR methods are most effective (Zmora et al., 2019) to assess them. In this experiment, both culture-dependent and -independent (qPCR-based) studies showed overgrowth of total anaerobic and aerobic bacteria, γ- and δ-Proteobacteria, Firmicutes, *Clostridium* sp., and *E. coli* in HFD and HFDC groups. Most of the notable changes were observed by restoration/overgrowth of healthier microbes such as *Bacteroides*, *Bifidobacterium*, and *Lactobacillus* group, as well as the ratio of Bacteroides *vs*. Firmicutes. The reactive metabolites synthesized by these healthier bacteria seemed to restrict the growth of *Clostridium* sp., *E. coli*, and other LPS-producing pathogenic bacteria in the gut. A consortium of food-grade microbes, oligosaccharides, and other nutraceuticals was already reported in fermented foods (Hor et al., 2021). Hence, its supplementation (in HFDT group) led to a significant change in overall gut microbiota structure and partially ameliorated HFD-induced dysbiosis. Experimentally, it was ascertained that polymerization (DP) of 3–4 (maltotriose and maltotetraose) in maltooligosaccharides and unsaturated fatty acids might expand growth of *Bifidobacterium, Akkermansia*, and *Lactobacillus* sp. and protect them from metabolic impairments (Zmora et al., 2019).

The ecological selection of food microbiota into the gut was demonstrated by polymerase chain reaction-denaturing gradient gel electrophoresis (PCR-DGGE) analysis. As *Lactobacillus* sp. (29.44%) was observed maximally in our rice fermented food, it was further targeted for DGGE analysis. Results highlighted that many species of LAB in rice fermented food were colonized in gut, which might potentially influence host biology and health. The detected microbes in fermented products were endophytic (root of *A. racemosus*) in origin and wild. The presence of anaerobic microbes in plant parts (root and shoot) was described by Kandel et al. (2017). Moreover, the consumption of *kimchi* and Parmigiano Reggiano cheese had also been reported to establish *Leuconostoc* strains and *Bifidobacterium mongoliense* in gut, respectively (Amato et al., 2021).

The levels of SCFAs such as acetate, propionate, and butyrate in intestine were elevated by more than twofold in HFDT compared to the HFD mice group (Table 3). The constituents of fermented rice might support growth of SCFAs producing specific microbes. These SCFAs are important metabolic intermediates of carbohydrate metabolism and modulate host’s systemic function. SCFAs have G-protein-coupled receptors (GPR) 43 and GPR41 [also called free fatty acid receptor (FFAR) 2 and FFAR3, respectively], which are ubiquitously present as nutritional sensors in most of the active cells. SCFAs are beneficial for host in multifaceted ways: (i) they are the prime energy source of colonocytes and support their structural integrity (precludes leaky gut) and prevent gut microbiota dysbiosis; (ii) they promote gluconeogenesis in hepatic and extrahepatic tissues and lower insulin resistance; (iii) they reduce appetite and food intake by stimulating the biosynthesis of leptin and gut hormones (e.g., glucagon-like peptide 1); (iv) they play an essential role in maintaining T_reg_ cell homeostasis; and (v) they stimulate the autonomic nervous system (ANS) (Morrison and Preston, 2016; Ríos-Covián et al., 2016; Al Bander et al., 2020). It had also been reported that SCFAs induce expression of PPAR-dependent switch from lipogenesis to lipolysis, thereby preventing obesity (den Besten et al., 2015).

Obesity is generally associated with cognitive impairment such as memory, anxiety, depression, and other social behaviors. The neuronal center of depression is the limbic system, and its associated area, i.e., hypothalamus, plays a crucial role in regulating energy balance and food intake. Recently, Vagena et al. (2019) has suggested that accumulation of fatty acid in hypothalamus (hypothalamic obesity) leads to an impairment of cAMP/PKA signaling cascade and thus contributes to depression. In obesity, hypertriglyceridemia-induced leptin resistance is also directly associated with cognitive dysfunctions (Del Rocío Sevilla-González et al., 2017; Milano et al., 2020). Frost et al. (2014) have suggested that acetate, an important constituent of SCFAs, can trigger an anorectic signal in hypothalamus by improving the glutamate–glutamine cycle and can increase biosynthesis of lactate and GABA (γ-aminobutyric acid), resulting in functional modulation of the brain–endocrine–adipose axis. By analyzing behavioral paradigms in mice, we demonstrated that HFD can influence learning-memory and anxiety-like depression phenotypes. Fermented rice (HFDT) might have antidepressant action as its mitigating behavioral alterations were induced by HFD. For the first time, our study demonstrated that rice fermented food not only alleviated obesity but also prevented its associated behavioral and psychological deficits through modulation of gut flora and their reactive metabolites.

**FIGURE 6:**
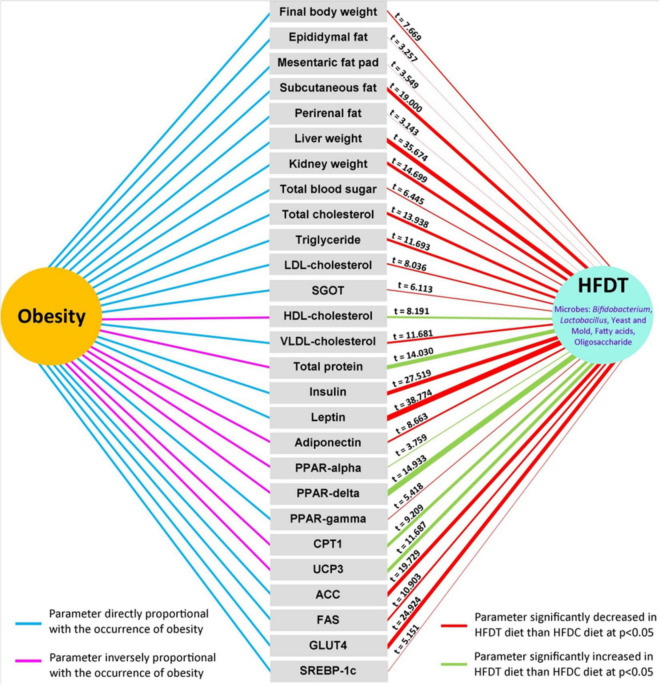
Overall significant impact of rice based fermented food on different physiological parameters directly and inversely related with obesity. Significant effects of fermented rice than non-fermented one are tested and values of *t*-score are given in each case. In the right hand side, red and green color connectors denoted significant decrease and increase of respective parameters upon supplementation of fermented diet than non-fermented one, and thickness of the connector implies relative magnitude of significance on *t*-score basis. In the left hand side, sky-blue and pink color connector indicates direct or inverse effect of parameters at onset of obesity.

In Figure 6, a summary of effects of HFDT on different aforementioned physiological indices has been represented and compared with the HFDC group. The documented relationship of different estimated physiological parameters with obesity is represented on the left side of the figure, where the blue linkage represents proportionate increment with obesity, while the pink line represents the opposite. On the right side of the figure, the effects of HFDT on all indices are represented after statistical comparison with the HFDC group. The red-colored linkage indicates that HFDT significantly minimizes the physiological level of respective parameters with respect to HFDC, while the green-colored linkage denotes the opposite (*p* < 0.05). The magnitudes of HFDT over HFDC effects are indicated by *t*-value on the respective linkage line and also on the basis of thickness of the latter. As a whole, the figure clearly represents overall impact of fermented diet on experimental mice model in relation to obesity in comparison with the unfermented counterpart.

## Conclusion

**FIGURE 7:**
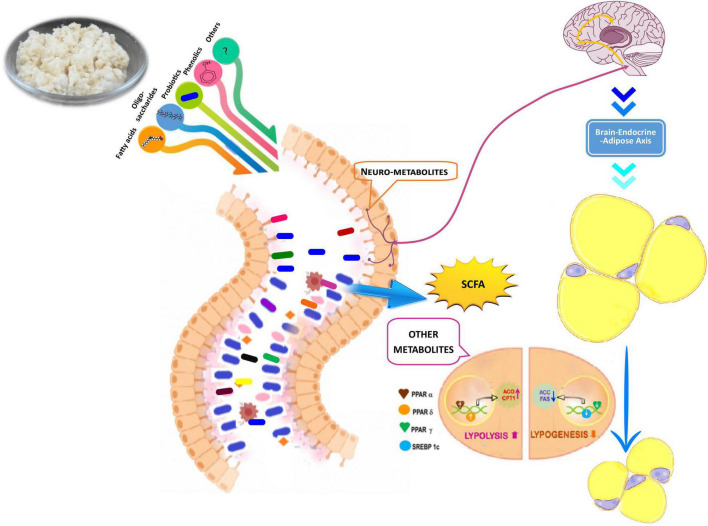
Hypothetical model for mode of action of rice based fermented food and its antiobesity propensity through colonization of gut flora, production of short chain fatty acids (SCFA) and other metabolites, lipolysis, lipogenesis process, and improvement of brain endocrine adipose axis.

The present study revealed that newly formulated fermented rice by using herbal starter of *A. racemosus* contained a consortium of food-graded microbes apart from its nutrients. This formulated food played a pivotal role in restoring gut microbial ecology, synchronized lipogenesis at the transcriptional level, improved glucose–insulin homeostasis, and also upgraded cognitive functions through a higher level of SCFA in diet-induced obese mice (Figure 7). The present study was accorded with the concept of diet–microbiota–host crosstalk that stated that a diet affected not only the relative or absolute abundance of gut flora but also their growth kinetics and microbial metabolites and thus tuned up overall homeostasis of host health (Zmora et al., 2019). The present study has several strengths and limitations. We have not employed metagenomics approaches to study the microbial absolute abundance (total microbial community) and metabolomics profile of gut. This experimental evidence supported the folklore use of herbal starters for the preparation of a variety of traditional foods and might create a new opportunity for the development of a rice-based functional food for alleviation of obesity and its associated disorders.

## Data Availability Statement

The datasets presented in this study can be found in online repositories. The names of the repository/repositories and accession number(s) can be found below: https://www.ncbi.nlm.nih.gov/, BioProject ID: PRJNA691916 and BioSample ID: SAMN17308527.

## Ethics Statement

The animal study was reviewed and approved by Institutional Ethics Committee (IEC) of Vidyasagar University.

## Author Contributions

PH, JM, SP, SH, MR, and DG performed experiments. SH performed statistical analysis. SC performed psychological behavior examination. MT analyzed the fatty acid profile. SSi, SD, KG, and KM analyzed the data. SSa, KG, DB, and KM prepared the manuscript. All authors contributed to the article and approved the submitted version.

## Conflict of Interest

The authors declare that the research was conducted in the absence of any commercial or financial relationships that could be construed as a potential conflict of interest.

## Publisher’s Note

All claims expressed in this article are solely those of the authors and do not necessarily represent those of their affiliated organizations, or those of the publisher, the editors and the reviewers. Any product that may be evaluated in this article, or claim that may be made by its manufacturer, is not guaranteed or endorsed by the publisher.

## Footnotes

1
https://www.mg-rast.org/
