# Supplementary material for: Antiobesity, Antihyperglycemic, and Antidepressive Potentiality of Rice Fermented Food Through Modulation of Intestinal Microbiota
Source: Front Microbiol. 2022 May 6;13:794503. doi: 10.3389/fmicb.2022.794503 (PMC9122802; doi:10.3389/fmicb.2022.794503)
Supplement: Supplementary file 1 [file Table_1.DOCX]

**Supplementary Table 1 |** Lists of target genes and their specific primers

| ***Lists of target genes and their specific primers used for gene expressions*** | | |
| --- | --- | --- |
| **Target gene** | **Forward primer (5'-3')** | **Reverse primer (5'-3')** |
| GAPDH | CAAGGCTGTGGGCAAGGTCA | AGGTGGAAGAGTGGGAGTTGCTG |
| PPARα | GTACGGTGTGTATGAAGCCATCTT | GCCGTACGCGATCAGCAT |
| PPARδ | GCCATATTCCCAGGCTGTC | CAGCACAAGGGTCATCTGTG |
| PPARγ | CTGGCCTCCCTGATGAATAAAG | GCGGTCTCCACTGAGAATAATG |
| ACO | GTGCAGCTCAGAGTCTGTCCAA | TACTGCTGCGTCTGAAAATCCA |
| CPT1 | GTGACTGGTGGGAGGAATAC | GAGCATCTCCATGGCGTAG |
| UCP3 | CCAGAGCATGGTGCCTTCGCT | CTCGTGTCAGCAGCAGTG |
| ANGPTL4 | AAAGAGGCTGCCCGAGAT | TCTCCCCAACCTGGAACA |
| ACC | ATGGGCGGAATGGTCTCTTTC | TGGGGACCTTGTCTTCATCAT |
| FAS | TGCTCCCAGCTGCAGGC | GCCCGGTAGCTCTGGGTGTA |
| TNFα | CCTCTTCTCATTCCTGCTTGT | GAGGTTGACTTTCTCCTGGTATG |
| GLUT4 | GGAAGGAAAAGGGCTATGCTG | GAGGAACCGTCCAAGAATGA |
| SREBP-1c | ACGGAGCCATGGATTGCACA | AAGGGTGCAGGTGTCACCTT |
|  |  |  |
| ***Lists of target bacteria and their 16S rRNA -targeted primers used for fecal microbiota analysis*** | | |
| **Target Bacteria** | **Forward primer (5'-3')** | **Reverse primer (5'-3')** |
| Universal | AAACTCAAAKGAATTGACGG | CTCACRRCACGAGCTGAC |
| Total bacteria | AGAGTTTGATCCTGGCTCAG | AAGGAGGTGWTCCARCC |
| *Proteobacteria* | GCTAACGCATTAAGTRYCCCG | GCCATGCRGCACCTGTCT |
| *Firmicutes* | GGAGYATGTGGTTTAATTCGAAGCA | AGCTGACGACAACCATGCAC |
| *Bacteroides* | GAGAGGAAGGTCCCCCAC | CGCTACTTGGCTGGTTCAG |
| *Lactobacillus* | GAGGCAGCAGTAGGGAATCTTC | GGCCAGTTACTACCTCTATCCTTCTTC |
| *Bifidobacterium* | CGCGTCYGGTGTGAAAG | CCCCACATCCAGCATCCA |
